# Endostar continuous versus intermittent intravenous infusion combined with chemotherapy for advanced NSCLC: a systematic review and meta-analysis including non-randomized studies

**DOI:** 10.1186/s12885-020-07527-4

**Published:** 2020-10-21

**Authors:** Bo Wang, Lu Xu, Qihuan Li, Sailimai Man, Cheng Jin, Lian Liu, Siyan Zhan, Yi Ning

**Affiliations:** 1 Department of Epidemiology, Meinian Institute of Health, Beijing, China; 2 Department of Epidemiology and Biostatistics, School of Public Health, Peking University, Beijing, China; 3 Department of Biostatistics, Meinian Institute of Health, Beijing, China; 4 State Key Laboratory of Translational Medicine and Innovative Drug Development, Nanjing, Jiangsu China; 5 Research Center of Clinical Epidemiology, Peking University Third Hospital, Beijing, China

**Keywords:** Recombinant human endostatin, Endostar, Non-small cell lung cancer, Systematic review, Meta-analysis, Non-randomized studies

## Abstract

**Background:**

Both intermittent intravenous (IIV) infusion and continuous intravenous (CIV) infusion of Endostar are widely used for NSCLC in China. We aimed to compare the efficacy and safety of CIV of Endostar versus IIV in combination with first-line chemotherapy for patients with advanced NSCLC.

**Methods:**

RCTs, NRCTs and cohort studies which compared CIV of Endostar with IIV in advanced NSCLC patients and reported efficacy or safety outcomes were eligible. Two reviewers independently screened records, extracted data and assessed risk of bias. Pooled risk ratios (RRs) with 95% confidence intervals were calculated using random effects meta-analysis for short-term efficacy and safety outcomes, and hazard ratios (HRs) for survival outcomes.

**Results:**

Finally nine studies involving 597 patients were included, containing two RCTs, three NRCTs and four cohort studies. For short-term efficacy, moderate quality of evidence showed that there were no significant differences between CIV of Endostar and IIV in objective response rate (ORR; RR 1.34, 95% CI 0.91–1.98, *P* = 0.14) and disease control rate (DCR; RR 1.11, 95% CI 0.94–1.30, *P* = 0.21). Very low quality of evidence indicated that CIV of Endostar significantly improved both overall survival (OS; HR 0.69, 95% CI 0.48–0.99, *P* = 0.046) and progression-free survival (PFS; HR 0.71, 95% CI 0.55–0.93, *P* = 0.01) compared with IIV. As for safety outcomes, moderate quality of evidence found that CIV of Endostar significantly reduced the risk of myelosuppression (RR 0.55, 95% CI 0.32–0.96, *P* = 0.03) and cardiovascular toxicity (RR 0.21, 95% CI 0.06–0.78, *P* = 0.02) compared with IIV.

**Conclusions:**

In advanced NSCLC, compared with IIV, CIV of Endostar had similar short-term efficacy, and substantially lower risk of myelosuppression and cardiovascular toxicity. Although very low quality of evidence supported the survival benefit of CIV compared with IIV, large RCTs with long-term follow-up are needed to demonstrate survival benefits. Caution should be given for off-label use of CIV of Endostar.

**Supplementary information:**

The online version contains supplementary material available at 10.1186/s12885-020-07527-4.

## Background

Lung cancer is a leading cause of cancer-related deaths on a global scale. A total of 2.09 million new cases of lung cancer occurred and 1.76 million patients died of lung cancer in 2018 [1]. Lung cancer imposes heavy economic burden worldwide. In China, the annual total cost of inpatients with lung cancer increased by an average of 16.15%, with the total expenditures of inpatients increasing from $2.16 billion in 1999 to $6.33 billion in 2005 [2]. Non-small cell lung cancer (NSCLC) accounts for around 80–85% of all cases with lung cancer. Adenocarcinoma (ADC) and squamous cell carcinoma (SqCC) are the two most common histological subtypes of NSCLC, approximately comprising 40–50% and 20–30% of all cases, respectively. Few patients with NSCLC are diagnosed at an early stage (stage I or II), at which point patients can be cured by surgical resection. Further, over 60% of patients suffered from locally advanced or metastatic lung cancer (stage III or IV) upon diagnosis [3].

For patients with advanced NSCLC, common first-line chemotherapy included docetaxel, gemcitabine, paclitaxel, vinorelbine plus cisplatin or carboplatin [4]. However, the efficacy of first-line platinum-containing therapy is limited and more effective therapies are needed. Endostatin, an angiogenesis inhibitor produced by hemangioendothelioma, was first discovered in 1997 [5]. Endostatin specifically inhibits endothelial proliferation and potently inhibits angiogenesis and tumor growth, which suggests the possibility of antiangiogenic therapy [6]. In 2005, a novel recombinant human endostatin, Recombinant Human Endostatin Injection (trade name: Endostar; code name: YH-16), was approved by China’s State Food and Drug Administration (SFDA) for the treatment of NSCLC [5]. Due to a nine amino acid sequence at the N-terminus (MGGSHHHHH), Endostar possessed better heat stability and proteolytic resistance compared with the endogenous protein [7].

Several studies have focused on the efficacy and safety of Endostar combined with chemotherapy versus chemotherapy alone [8–11]. A phase II, multicenter, randomized, double-blind, placebo-controlled study compared the efficacy and safety of Endostar plus paclitaxel-carboplatin (TC regimen) with placebo plus TC in advanced NSCLC patients [8]. Endostar plus TC seemed to increase objective response rate (ORR; 39.3% versus 23.0%, *P* = 0.078) and disease control rate (DCR; 90.2% versus 67.2%, *P* = 0.004). However, there was no statistically significant difference in progression-free survival (PFS) or overall survival (OS) as well as the incidence of adverse events or serious adverse events between the two groups. According to a phase III, multicenter, randomized, double-blind, placebo-controlled study [9], Endostar combined with vinorelbine-cisplatin (NP regimen) enhanced the efficacy of NP regimen. In comparison with placebo plus NP, Endostar plus NP improved ORR (35.4% versus 19.5%, *P* = 0.0003) and DCR (73.3% versus 64.0%, *P* = 0.035). Furthermore, Endostar plus NP prolonged time to progression (TTP; 6.3 months versus 3.6 months, *P* < 0.001), OS (13.8 months versus 9.8 months, *P* < 0.0001) and increased quality of life score (QoL score; 54.4 ± 3.7 versus 53.4 ± 5.9, *P* = 0.0155). Additionally, Endostar did not increase the incidence of grade 3/4 adverse events. A meta-analysis [10] of 15 RCTs also confirmed that the combination of Endostar and platinum-containing two-drug chemotherapy significantly increased ORR and DCR, prolonged TTP, improved one-year survival rate and QoL, and did not increase the risk of adverse events compared with platinum-containing two-drug chemotherapy alone. Another meta-analysis [11] of prospective clinical trials found that Endostar combined with vinorelbine plus cisplatin chemotherapy (NP regimen) increased ORR and improved one-year survival rate of patients with advanced NSCLC, compared with NP regiment alone.

Traditionally, Endostar is administrated by intermittent intravenous (IIV) infusion for 3–4 h per day during a 14-day period, which unavoidably causes more inconvenience, affects quality of life and may reduce patient compliance. Besides, IIV also increased intravenous admixture workload of medical staff and the use of hospital beds. Since 2010, continuous intravenous (CIV) infusion via an infusion pump, which is able to deliver a variety of solutions at a constant rate for days and even weeks, has been introduced and widely off-label used in clinical practice in China. It has been argued that with this new modality for delivering a continuous infusion for days, an effective concentration of drug will be maintained and thereby the efficacy will be enhanced [12]. To our knowledge, however, the efficacy and safety of CIV versus IIV in combination with first-line chemotherapy in treating patients with advanced NSCLC have not been systematically evaluated yet. This systematic review and meta-analysis aimed to compare the two administration strategies in terms of efficacy and safety.

## Method

### Search strategy

We searched the following sources: 1) Electronic databases including Pubmed, Embase, CENTRAL (Cochrane Central Register of Controlled Trials), CDSR (Cochrane Database of Systematic Review), CINAHL (the Cumulative Index to Nursing and Allied Health Literature), PsycINFO and SinoMed; 2) Clinical trial registries including ClinicalTrials.gov (www.clinicaltrials.gov) and ChiCTR (Chinese Clinical Trial Registry); 3) Citation databases including Science Citation Index Expanded and CSCD (Chinese Science Citation Database); 4) CPCI (Conference proceedings including Conference Proceedings Citation Index) and ASCO Meeting Library; 5) Reference lists of all relevant guidelines, reviews and finally included articles; 6) Consultation with related researchers.

All databases were searched from inception to May 14, 2020 without language restriction. The keywords for the search strategy included “Endostar”, “recombinant human endostatin”, “Rh-endostatin” and “YH-16”.

### Inclusion and exclusion criteria

Eligible study types included randomized controlled trials (RCTs), non-randomized controlled trials (NRCTs) and cohort studies. Single-arm trials, case-control studies, controlled before-and-after studies, historically controlled studies, interrupted time series studies, cross-sectional studies, case series, commentaries, editorials, letters, reviews, case reports and experimental studies were excluded. Eligible participants were patients with pathologically confirmed stage III or IV NSCLC, either for initial treatment or retreatment. All pathological types were eligible to be included. Eligible interventions/controls were CIV versus IIV of Endostar in combination with first-line chemotherapy, with no limits in the dose or duration of Endostar treatment. Studies comparing Endostar combined with first-line chemotherapy versus chemotherapy alone, or those comparing Endostar combined with chemotherapy with placebo, were excluded. The study outcomes should include any of the following: 1) overall survival (OS); 2) progression-free survival (PFS); 3) ORR; 4) DCR; 5) TTP; 6) adverse events. ORR and DCR were evaluated by response evaluation criteria in solid tumors (RECIST) [13].

For studies with multiple publications or studies with same results published in different journals, the most comprehensive one with the largest sample size was chosen. Two investigators (LX and QL) independently screened the titles and abstracts of citations retrieved, and the full texts of potentially eligible articles were obtained and further assessed for final inclusion. Disagreements were resolved through consensus or consultation with a senior investigator (BW).

### Data extraction and risk of bias assessment

We collected information regarding study characteristics (study design, inclusion criteria, patient characteristics, sample size, intervention and control details, outcomes, and main results) and methodological characteristics (participants recruitment, randomization, assignment concealment, blinding, intervention compliance, follow-up, outcome evaluation, statistical analysis).

Due to the variability of study designs included (i.e., RCTs, NRCTs and cohort studies), Downs and Black Checklist was used to assess risk of bias in each included study [14, 15]. The items contained mainly address the following specific domains: 1) participants recruitment; 2) randomization; 3) allocation concealment; 4) blinding; 5) intervention compliance; 6) follow-up; 7) outcome assessment; 8) statistical analysis. The included 13 items can be seen in Table S1 (see Additional file 1). The possible score assigned for each item was 0 (no or unclear) and 1 (yes). The overall score ranged from 0 to 13, with 0–5 standing for very serious risk of bias, 6–7 for serious risk of bias, and 8–13 for acceptable risk of bias.

Data extraction and quality evaluation were conducted by two investigators (LX and QL) in duplicate as well. Any disagreement was negotiated by the two investigators or judged by a third senior investigator (BW). Furthermore, Grading of Recommendations Assessment, Development, and Evaluation (GRADE) [16], which includes 5 aspects (study limitation, indirectness, inconsistency, imprecision and publication bias), was utilized to evaluate the quality of evidence contributing to each study outcome.

### Statistical analysis

For survival outcomes (OS and PFS), hazard ratios (HRs) was chosen as the effect measure, and inverse variance method was employed to conduct the meta-analysis. For dichotomous outcomes (ORR, DCR and adverse events), risk ratios (RRs) was selected as effect measure and Mantel-Haenszel method was used for meta-analysis. In current systematic review, all the analysis were performed by using random-effect model, and were implemented and presented according to study designs (it was not appropriate to combine results from different study designs). For studies in which HR was not provided for survival outcomes, we estimated HR and its 95% CI from Kaplan-Meier curve according to the method by Tierney et al. [17]. Meanwhile, sensitivity analysis for this approximate estimation was undertaken.

Chi-square test was used to evaluate whether there was statistically significant heterogeneity among studies, and the significance level was set to 0.10. Heterogeneity between studies was also assessed by I^2^ statistic, which estimates the percentage of total variation across studies due to true between-study differences rather than chance. If there was significant heterogeneity (*P* < 0.10 and I^2^ ≥ 50%), sensitivity analysis was also carried out to evaluate the robustness of results by excluding obviously outlying studies. As funnel plots should only be used if there are at least ten studies included in the meta-analysis, Harbord’ test and Egger’s test were implemented to identify publication bias for dichotomous and survival outcomes, respectively. Publication bias could not be detected for outcomes with less than two studies included, as the statistical tests are reliable with at least three studies included in the analysis. Statistical analyses were done using Stata 15 (Stata Corp., College Station, TX, USA).

## Results

### Study characteristics and risk of Bias

**Fig. 1:**
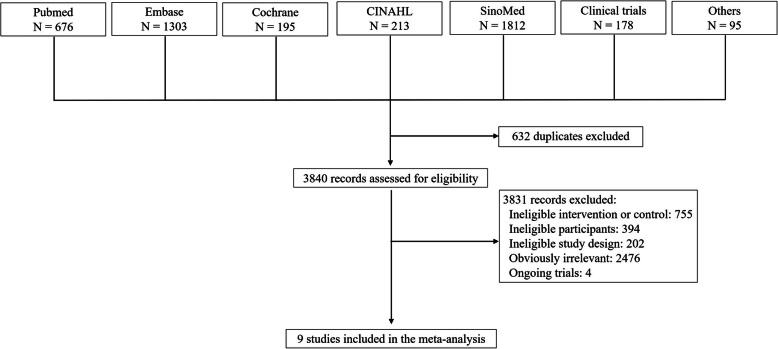
Selection of eligible studies

Figure 1 showed the selection of eligible studies. After removing 632 duplicates, 3840 records were retrieved from literature search. Three thousand eight hundred thirty-one records were further excluded as they did not meet the inclusion criteria. Finally, nine studies were identified as eligible for inclusion in this systematic review, including four cohort studies [18–21] and two RCTs [22, 23] and three NRCTs [24–26].

Table 1 showed the characteristics of included studies. All nine studies were conducted in China, and were published between 2013 and 2019. A total of 597 advanced NSCLC participants were included (CIV: 300 and IIV: 297), with ages ranging from 24 to 78 years. The histological types of NSCLC included both SqCC and ADC in five studies [20–24], only SqCC in one study [18], and were unknown in three studies [19, 25, 26]. TNM staging of advanced NSCLC included both stage III and IV in six studies [18–20, 22–24], only stage IV in one study [26], and were not reported in two studies [21, 25]. Included studies were relatively small, with sample sizes ranging between 28 and 116. In each treatment cycle, Endostar was given at a daily dose of 7.5 mg/m^2^/d, for 3–4 h from day 1 to 14, in the IIV group. In the CIV group, Endostar was administered via an infusion pump [18, 21–26] or a mini-osmotic pump [19, 20], at a dose of 210 mg with a speed of 1.8–10 mL/h in seven studies [19–23, 25, 26], a dose of 225 mg with a speed of 2 mL/h in one study [24], and a dose of 135 mg with a speed of 11 mL/h in one study [18]. In all included studies, Endostar was administered in combination with first-line chemotherapy, and no surgery or radiotherapy was implemented. ORR, DCR and adverse events were reported in all nine studies, OS in two studies [18, 19], and PFS in four studies [18–21].

**Table 1:** Study characteristics of included studies

Study	Study design	Participants	Sample size (M/F)	Intervention/control ^a^	Outcomes
Yao et al.	Cohort study	45–75 years old (mean: 63); all patients with SqCC; stage III B 20 cases/stage IV 51 cases	71 (69/2); CIV: 48 (47/1); IIV: 23 (22/1)	CIV: Endostar by infusion pump (15 mg/d, 11 ml/h) from day 0 to 8, combined with GP chemotherapy (gemcitabine and cisplatin); IIV: Endostar by intravenous infusion (15 mg/d, 4 h/d) from day 0 to 13, combined with GP chemotherapy (gemcitabine and cisplatin)	Efficacy: OS, PFS, ORR, DCR; safety: leukopenia, neutropenia, thrombocytopenia, anemia, hyponatremia, Transaminase elevation, laryngeal hemorrhage, hypertension, fatigue, nausea & vomiting, constipation, diarrhea, papule, purulent herpes, fever, thromboembolism
Li et al.	Cohort study	41–70 years old; all patients with NSCLC; stage III B 34 cases/stage IV 82 cases	116 (77/39); CIV: 58 (40/18); IIV: 58 (37/21)	CIV: Endostar by mini-osmotic pump (210 mg, 5 ml/h) from day 1 to 10, combined with docetaxel, gemcitabine, pemetrexed, cisplatin, nedaplatin, etc.; IIV: Endostar by intravenous infusion (15 mg/d, 4 h/d) from day 1 to 14, combined with docetaxel, gemcitabine, pemetrexed, cisplatin, nedaplatin, etc.	Efficacy: OS, PFS, ORR, DCR; safety: neutropenia, anemia, thrombocytopenia, hemorrhage, nausea & vomiting, mucositis, constipation, diarrhea, transaminase elevation, total bilirubin elevation, blood creatinine elevation, fever, rash, fatigue, pain, allergy, peripheral neurotoxicity, alopecia, arrhythmia
Cheng et al.	Cohort study	33–78 years old (mean: 59.5); SqCC 21 cases/ADC: 36 cases/other types 12 cases; stage III 7 cases/stage IV 54 cases/postoperative recurrence 8 cases	69 (50/19); CIV: 20 (13/7); IIV: 49 (37/12)	CIV: Endostar by mini-osmotic pump (105 mg/m^2^, 10 ml/h) from day 1 to 5, combined with first-line chemotherapy (gemcitabine and platinum, pemetrexed and platinum, or paclitaxel and platinum); IIV: Endostar by intravenous infusion (7.5 mg/m^2^/d, 4 h/d) from day 1 to 14, combined with first-line chemotherapy (gemcitabine and platinum, pemetrexed and platinum, or paclitaxel and platinum)	Efficacy: PFS, ORR, DCR; safety: Neutropenia, anemia, thrombocytopenia, arrhythmia, myocardial ischemia, neurotoxicity, rash, transaminase elevation, vomiting, infection, nausea, deep vein thrombosis, hemorrhage
Zhu et al.	Cohort study	33–75 years old (median: 58); SqCC 16 cases/ADC 45 cases/other types 3 cases; all patients with advanced NSCLC	64 (46/18); C: 33; IIV: 31	CIV: Endostar by infusion pump (210 mg, 3 ml/h) from day 1 to 7, combined with AP chemotherapy (pemetrexed and cisplatin) or TP (paclitaxel and cisplatin); IIV: Endostar by intravenous infusion (15 mg/d, 4 h/d) from day 1 to 14, combined with AP chemotherapy (pemetrexed and cisplatin) or TP (paclitaxel and cisplatin)	Efficacy: PFS, ORR, DCR; safety: myelosuppression, liver dysfunction, gastrointestinal reaction, cardiovascular toxicity, peripheral neurotoxicity, hemoptysis
Pang et al.	RCT	41–76 years old (mean: 61); SqCC 33 cases/ADC 45 cases/AdSqCC 3 cases/LCC 3 cases; stage III B 32 cases/stage IV 52 cases	84 (48/36); CIV: 42 (23/19); IIV: 42 (25/17)	CIV: Endostar by infusion pump (7.5 mg/m^2^/d, 5 ml/h) from day 1 to 7, combined with GP chemotherapy (gemcitabine and cisplatin); IIV: Endostar by intravenous infusion (7.5 mg/m^2^/d, 3–4 h/d) from day 1 to 14, combined with GP chemotherapy (gemcitabine and cisplatin)	Efficacy: ORR, DCR; safety: cardiovascular toxicity, myelosuppression, nausea & vomiting, liver dysfunction, neurotoxicity, fatigue, diarrhea
Wen and Chen	RCT	24–76 years old (median: 58); SqCC 39 cases/ADC 28 cases/AdSqCC 4 cases; stage III B 48 cases/stage IV 23 cases; initial treatment 46 cases/retreatment 25 cases	71 (45/26); CIV: 37; IIV: 34	CIV: Endostar by infusion pump (7.5 mg/m^2^/d, 10 ml/h) from day 1 to 14, combined with TC chemotherapy (paclitaxel and carboplatin); IIV: Endostar by intravenous infusion (7.5 mg/m^2^/d, 3–4 h/d) from day 1 to 14, combined with TC chemotherapy (paclitaxel and carboplatin)	Efficacy: ORR, DCR; safety: cardiovascular toxicity, myelosuppression, nausea & vomiting, alopecia, muscle & joint soreness
Kahaerjiang et al.	NRCT	34–75 years old (median: 51.5); SqCC 16 cases/ADC 12 cases; stage III 12 cases/stage IV 16 cases	28 (20/8); CIV: 14; IIV: 14	CIV: Endostar by infusion pump (225 mg, 2 ml/h) from day 1 to 15, combined with NP chemotherapy (vinorelbine and cisplatin); IIV: Endostar by intravenous infusion (7.5 mg/m^2^/d, 3–4 h/d) from day 1 to 14, combined with NP chemotherapy (vinorelbine and cisplatin)	Efficacy: ORR, DCR; safety: leukopenia, neutropenia, anemia, nausea & vomiting, cardiovascular toxicity
Tang et al.	NRCT	26–78 years old; all patients with advanced NSCLC	54 (34/20); CIV: 28; IIV: 26	CIV: Endostar by intravenous infusion (7.5 mg/m^2^, 4 h) on day 1, Endostar by infusion pump (195 mg, 1.8 ml/h) from day 2 to 8, combined with GP chemotherapy (gemcitabine and cisplatin); IIV: Endostar by intravenous infusion (7.5 mg/m^2^/d, 4 h/d) from day 1 to 14, combined with GP chemotherapy (gemcitabine and cisplatin)	Efficacy: ORR, DCR; safety: cardiovascular toxicity, myelosuppression, nausea & vomiting, alopecia
Meng	NRCT	25–70 years old (mean: 57); all patients with stage IV NSCLC	40 (28/12); CIV: 20 (13/7); IIV: 20 (15/5)	CIV: Endostar by intravenous infusion (7.5 mg/m^2^, 4 h) on day 1, Endostar by infusion pump (195 mg, 1.8 ml/h) from day 2 to 8, combined with GP chemotherapy (gemcitabine and cisplatin); IIV: Endostar by intravenous infusion (7.5 mg/m^2^/d, 4 h/d) from day 1 to 14, combined with GP chemotherapy (gemcitabine and cisplatin)	Efficacy: ORR, DCR; safety: cardiovascular toxicity, myelosuppression, gastrointestinal reaction

^a^ The treatment cycle of all included studies was 21 days. In all studies patients were treated for at least two cycles, except for one study [21] in which patients were treated for at least one cycle

The results of risk of bias assessment can be found in Table S2 (see Additional file 1). All studies were not blinded to patients or outcome measurers. No results were based on data dredging, as no retrospective subgroup analyses were reported. In all included studies, the analyses adjusted for different lengths of follow-up of patients by survival analysis or the follow-up was the same for all participants, and appropriate statistical methods were used to assess the main outcomes. Compliance with interventions was reliable in each study, as there was no non-compliance with the allocated treatment or contamination of intervention groups. In all nine studies, participants in different intervention groups were recruited from the same population and over the same period of time, and the main outcome measures used were valid and reliable. Although patients were randomized to intervention groups in two RCTs [22, 23], we were unable to judge whether allocation was concealed from both patients and health care staff. Baseline characteristics were comparable between intervention groups in all studies but one [24], in which comparability between groups was not reported and no adjustment for confounding was conducted. None of the nine studies reported any losses of patients to follow-up. Therefore, all included studies were evaluated as with acceptable risk of bias.

### Efficacy outcomes

**Fig. 2:**
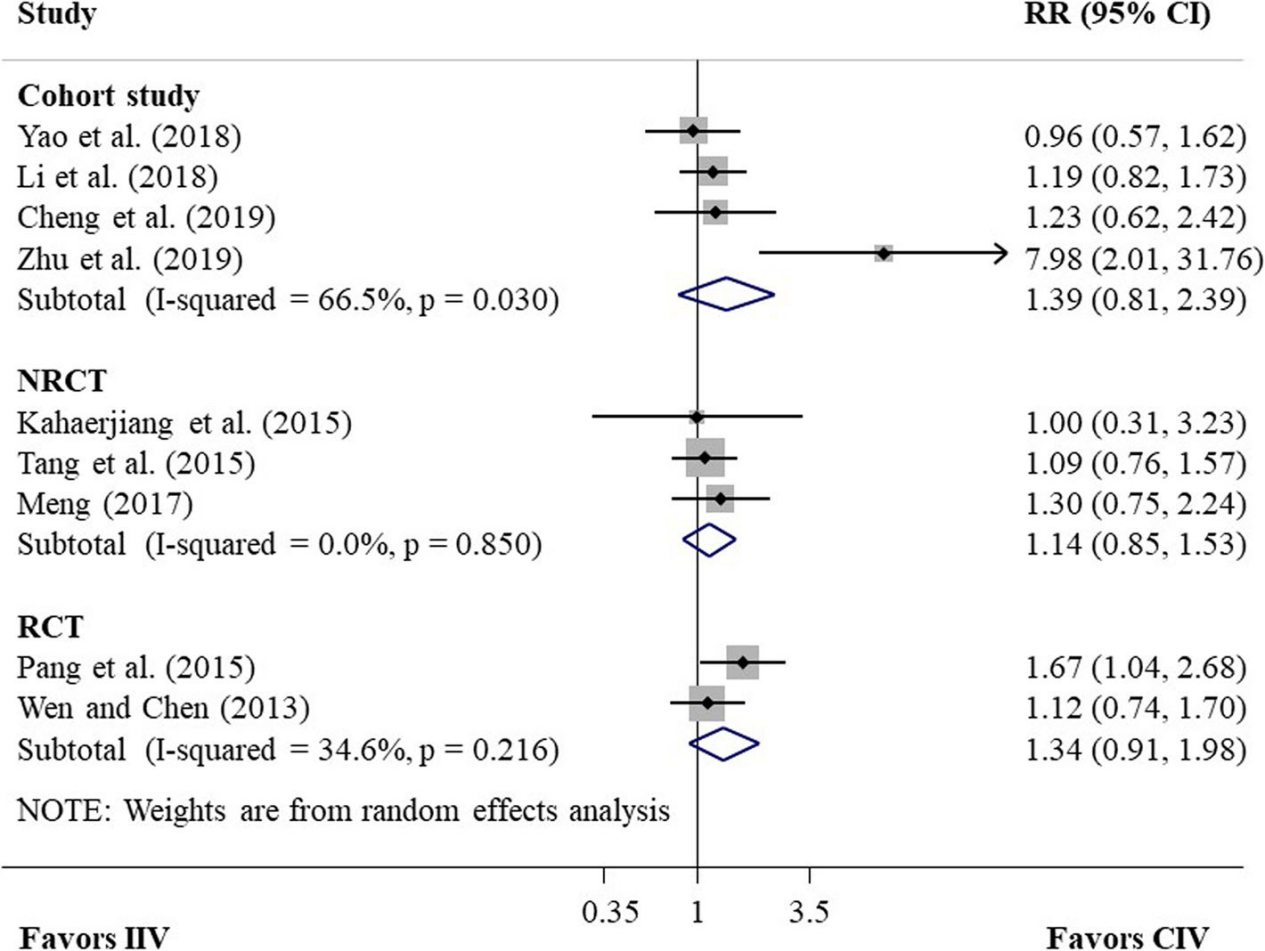
Forest plot of pooled RR of ORR for CIV compared with IIV from meta-analysis of studies with different study designs

Table 2 summarized pooled results for short-term efficacy outcomes and survival outcomes between CIV and IIV of Endostar, including corresponding evidence quality in each outcome. Two RCTs [22, 23], involving 155 NSCLC patients, reported the RR of ORR for CIV compared with IIV, and the pooled RR of ORR was 1.34 (95% CI 0.91–1.98, *P* = 0.14; heterogeneity test, I^2^ = 35%, *P* = 0.22; random-effects meta-analysis; Fig. 2). Statistical test for publication bias was not performed due to less than two included studies (similarly hereinafter). The quality of evidence was moderate. Three NRCTs [24–26], including a total of 122 NSCLC patients, reported the RR of ORR and the pooled RR was 1.14 (95% CI 0.85–1.53, *P* = 0.37; heterogeneity test, I^2^ = 0%, *P* = 0.85; random-effects meta-analysis; Fig. 2), with low quality of evidence. Harbord’s test did not indicate publication bias (*P* = 0.99). Four cohort studies [18–21], including 317 patients, reported RR of ORR and the pooled RR was 1.39 (95% CI 0.81–2.39, *P* = 0.23; heterogeneity test, I^2^ = 67%, *P* = 0.03; random-effects meta-analysis; Fig. 2), with very low quality of evidence. Harbord’s test did not suggested publication bias (*P* = 0.43). Sensitivity analysis removing one obvious outlier study [21] identified no significant heterogeneity among remaining studies and did not materially change the results.

**Table 2:** The pooled results and evidence quality in efficacy outcomes

Outcome	RCT		NRCT		Cohort study	
	Estimate (95% CI)	Evidence quality ^a^	Estimate (95% CI)	Evidence quality ^a^	Estimate (95% CI)	Evidence quality ^a^
**Short-term outcome**						
ORR	RR 1.34 (0.91–1.98)	Moderate	RR 1.14 (0.85–1.53)	Low	RR 1.39 (0.81–2.39)	Very low
DCR	RR 1.11 (0.94–1.30)	Moderate	RR 1.09 (0.91–1.30)	Low	RR 1.07 (0.94–1.21)	Very low
**Survival outcome**						
OS	–	–	–	–	HR 0.68 (0.42–0.94) ^*^	Very low
PFS	–	–	–	–	HR 0.71 (0.51–0.90) ^*^	Very low

^a^ The evidence quality was evaluated by GRADE. Moderate: we are moderately confident in the effect estimate (the true effect is likely to be close to the estimate of the effect, but there is a possibility that it is substantially different); Low: our confidence in the effect estimate is limited (the true effect may be substantially different from the estimate of the effect); Very low: we have very little confidence in the effect estimate (the true effect is likely to be substantially different from the estimate of effect)

^*^ A statistically significant difference exists (*P* < 0.05)

**Fig. 3:**
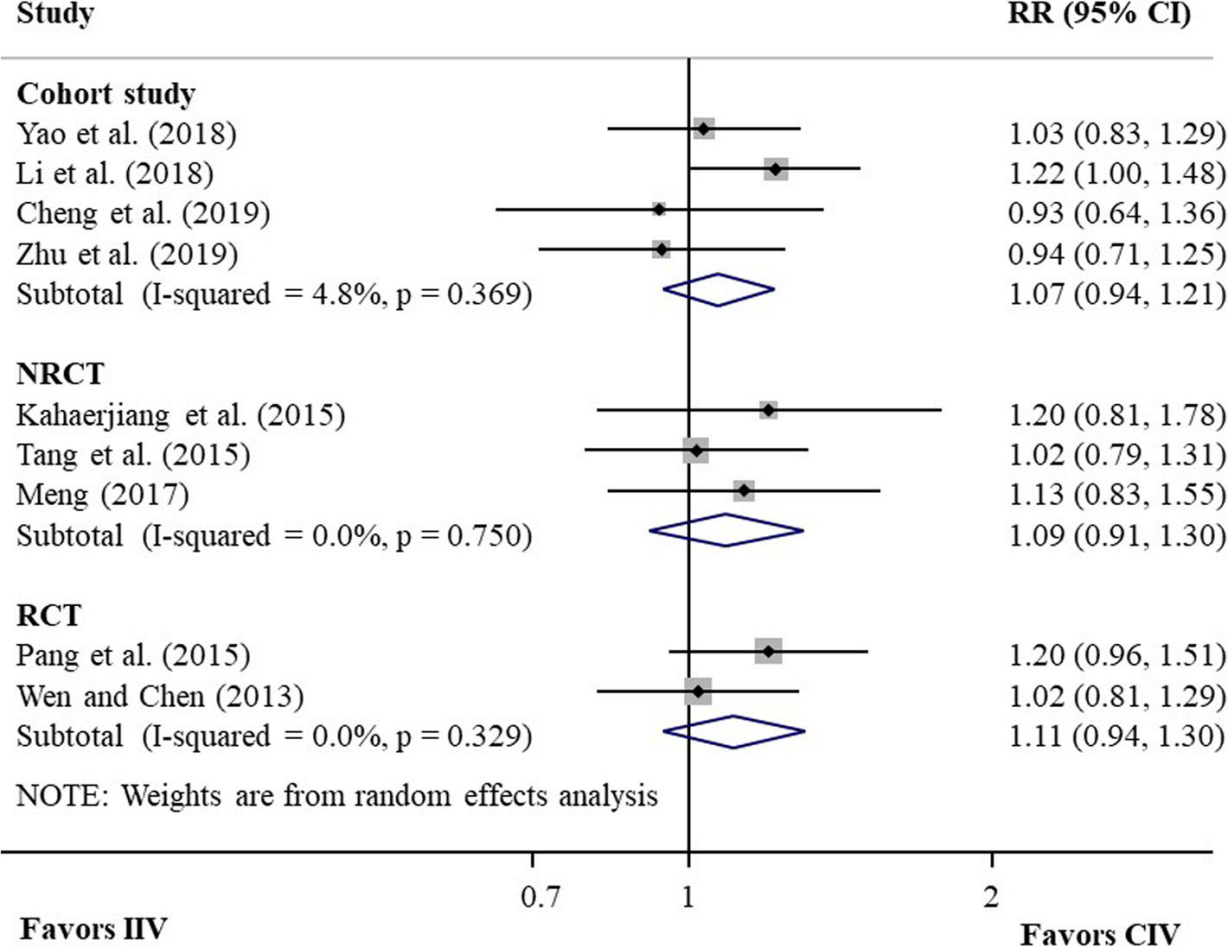
Forest plot of pooled RR of DCR for CIV compared with IIV from meta-analysis of studies with different study designs

Two RCTs [22, 23], involving 155 NSCLC patients, reported the RR of DCR for CIV compared with IIV, and the pooled RR of DCR was 1.11 (95% CI 0.94–1.30, *P* = 0.21; heterogeneity test, I2 = 0%, *P* = 0.33; random-effects meta-analysis; Fig. 3). The quality of evidence was moderate. Three NRCTs [24–26], including a total of 122 NSCLC patients, reported the RR of DCR and the pooled RR was 1.09 (95% CI 0.91–1.30, *P* = 0.35; heterogeneity test, I2 = 0%, *P* = 0.75; random-effects meta-analysis; Fig. 3), with low quality of evidence. Harbord’s test did not find publication bias (*P* = 0.17). Four cohort studies [18–21], including 317 patients, reported RR of DCR and the pooled RR was 1.07 (95% CI 0.94–1.21, *P* = 0.30; heterogeneity test, I2 = 5%, *P* = 0.37; random-effects meta-analysis; Fig. 3), with very low quality of evidence. Harbord’s test suggested no publication bias (*P* = 0.18).

**Fig. 4:**
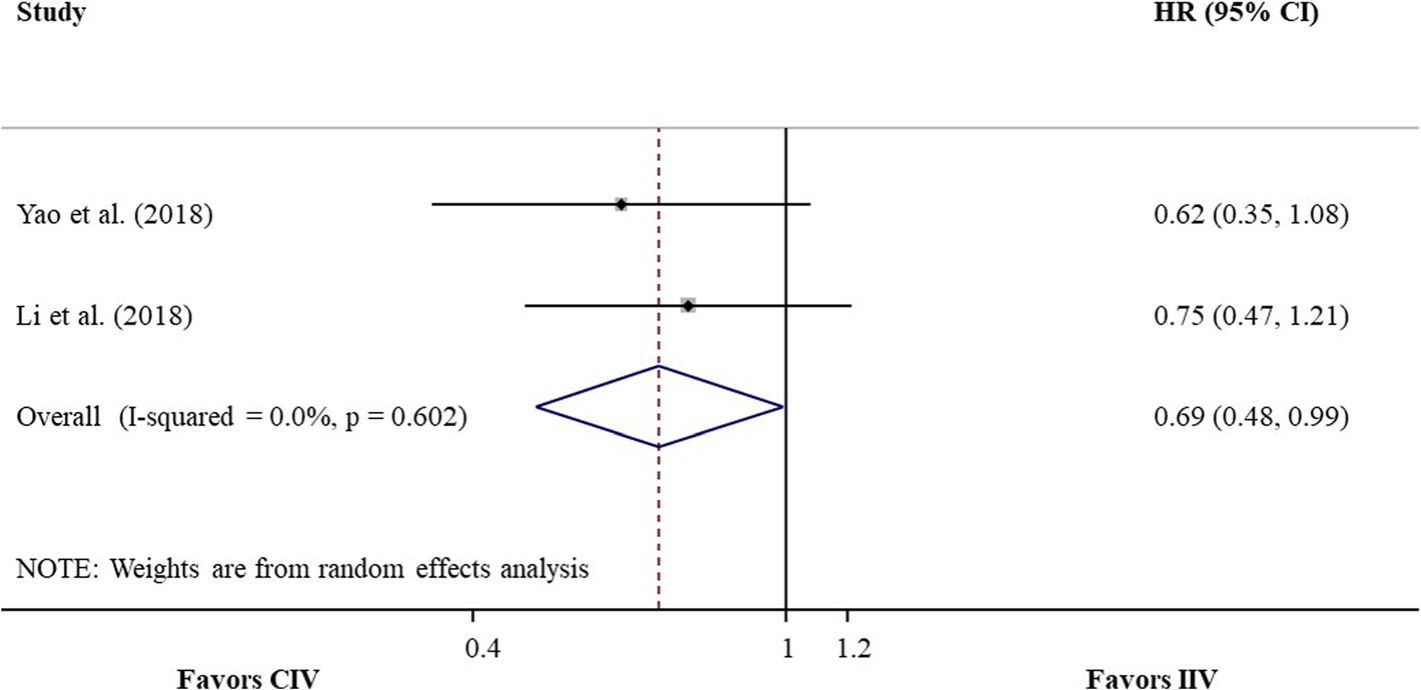
Forest plot of pooled HR of OS for CIV compared with IIV from meta-analysis of cohort studies

**Fig. 5:**
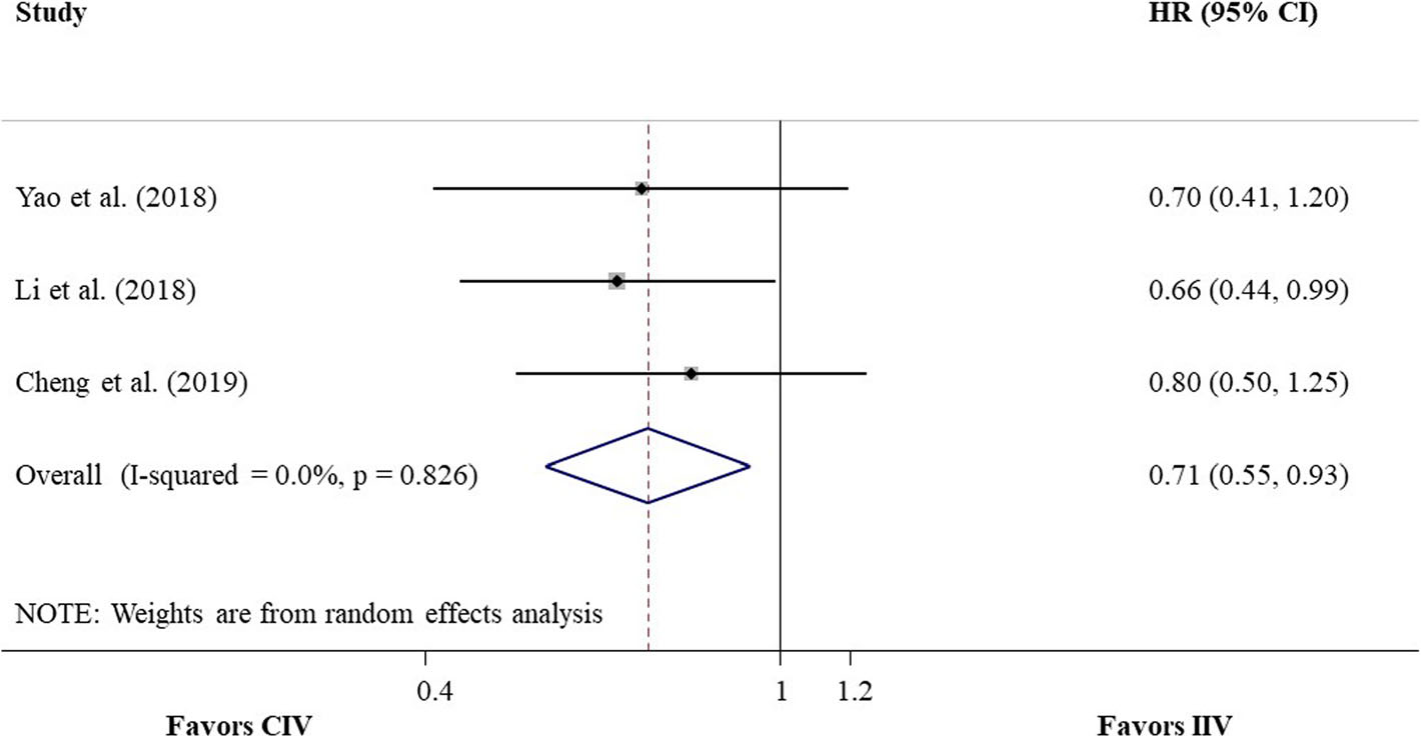
Forest plot of pooled HR of PFS for CIV compared with IIV from meta-analysis of cohort studies

Two cohort studies [18, 19], involving 187 patients, provided the HR or Kaplan-Meier curve of OS for CIV compared with IIV, and the pooled HR was 0.69 (95% CI 0.48–0.99, *P* = 0.046; heterogeneity test, I^2^ = 0%, *P* = 0.60; random-effects meta-analysis; Fig. 4), with very low quality of evidence. Three cohort studies [18–20], including a total of 256 patients, provided the HR or Kaplan-Meier curve of PFS for CIV compared with IIV, and the pooled HR was 0.71 (95% CI 0.55–0.93, *P* = 0.01; heterogeneity test, I2 = 0%, *P* = 0.83; random-effects meta-analysis; Fig. 5), with very low quality of evidence. No publication bias was suggested by Egger’s test (*P* = 0.81). For both OS and PFS, sensitivity analysis, which excluded studies with approximate estimation of HR, did not materially change the direction and CIs of the results.

### Safety outcomes

Table 3 exhibited the results on safety outcomes between CIV and IIV, based on meta-analysis of studies by different study designs and corresponding evidence quality in each safety outcome. The pooled results found that compared with IIV, CIV reduced the risk of myelosuppression (RR 0.55, 95% CI 0.32–0.96, *P* = 0.03; heterogeneity test, I^2^ = 58%, *P* = 0.12; random-effects meta-analysis; moderate quality of evidence) and cardiovascular toxicity (RR 0.21, 95% CI 0.06–0.78, *P* = 0.02; heterogeneity test, I^2^ = 0%, *P* = 0.59; random-effects meta-analysis; moderate quality of evidence). In addition, one cohort study [18] reported that CIV reduced the risk of laryngeal hemorrhage in comparison to IIV (RR 0.27, 95% CI 0.09–0.84, P = 0.02; very low quality evidence). One NRCT [25] reported that CIV reduced the risk of alopecia (RR 0.65, 95% CI 0.42–1.00, *P* = 0.05), however, this was not confirmed by one RCT (RR 0.95, 95% CI 0.82–1.09, *P* = 0.45). For all the other adverse events, no statistically significant differences were found between CIV and IIV of Endostar, and Harbord’s test suggested no publication bias for those outcomes with at least three studies included.

**Table 3:** The pooled results and evidence quality in safety outcomes

Outcome	RCT		NRCT		Cohort study	
	Estimate (95% CI)	Evidence quality ^a^	Estimate (95% CI)	Evidence quality ^a^	Estimate (95% CI)	Evidence quality ^a^
**Myelosuppression**	RR 0.55 (0.32–0.96) ^*^	Moderate	RR 0.38 (0.20–0.72) ^*^	Low	RR 1.11 (0.59–2.10)	Very low
Grade 3/4 myelosuppression	RR 0.70 (0.29–1.66)	Low	RR 0.20 (0.01–3.92)	Very low	RR 1.25 (0.30–5.15)	Very low
Leukopenia	–	–	RR 0.44 (0.18–1.11)	Very low	RR 1.12 (0.79–1.58)	Very low
Grade 3/4 leukopenia	–	–	–	–	RR 0.80 (0.33–1.93)	Very low
Neutropenia	–	–	RR 0.55 (0.28–1.06)	Very low	RR 1.04 (0.89–1.22)	Very low
Grade 3/4 neutropenia	–	–	–	–	RR 0.96 (0.64–1.43)	Very low
Anemia	–	–	RR 0.43 (0.14–1.33)	Very low	RR 1.05 (0.92–1.20)	Very low
Grade 3/4 anemia	–	–	–	–	RR 0.95 (0.46–1.97)	Very low
Thrombocytopenia	–	–	–	–	RR 1.15 (0.74–1.79)	Very low
Grade 3/4 thrombocytopenia	–	–	–	–	RR 1.30 (0.49–3.47)	Very low
Hemorrhage	–	–	–	–	–	–
Grade 3/4 hemorrhage	–	–	–	–	–	–
Laryngeal hemorrhage	–	–	–	–	RR 0.27 (0.09–0.84) ^*^	Very Low
**Cardiovascular toxicity**	RR 0.21 (0.06–0.78) ^*^	Moderate	RR 0.15 (0.02–1.20)	Very low	–	–
Cardiotoxicity	–	–	RR 0.26 (0.06–1.13)	Very low	–	–
**Gastrointestinal Reaction**	–	–	RR 0.83 (0.30–2.29)	Very low	RR 1.25 (0.30–5.15)	Very low
Grade 3/4 gastrointestinal response	–	–	RR 0.67 (0.12–3.57)	Very low	–	–
Nausea & vomiting	RR 0.88 (0.51–1.51)	Low	RR 0.58 (0.25–1.36)	Very low	RR 0.97 (0.72–1.31)	Very low
Grade 3/4 nausea & vomiting	RR 1.00 (0.38–2.60)	Low	RR 1.00 (0.16–6.14)	Very low	RR 1.25 (0.35–4.42)	Very low
Nausea	–		–	–	RR 1.17 (0.32–4.28)	Very low
Vomiting	–		–	–	RR 1.23 (0.63–2.40)	Very low
Diarrhea	RR 1.43 (0.60–3.40)	Low	–	–	RR 0.60 (0.18–1.96)	Very low
Constipation	–		–	–	RR 0.90 (0.53–1.54)	Very low
**Alopecia**	RR 0.95 (0.82–1.09)	Moderate	RR 0.65 (0.42–1.00) ^*^	Very low	RR 0.78 (0.31–1.95)	Very low
**Neurotoxicity**	RR 0.80 (0.23–2.77)	Low	–	–	–	–
Peripheral neurotoxicity	–		–	–	RR 0.58 (0.14–2.33)	Very low
**Liver dysfunction**	RR 0.71 (0.39–1.29)	Low	–	–	RR 0.63 (0.20–2.01)	Very low
Grade 3/4 liver dysfunction	RR 0.60 (0.15–2.35)	Low	–	–	–	
Transaminase elevation	–		–	–	RR 1.48 (0.84–2.62)	Very low
**Fatigue**	RR 1.14 (0.87–1.50)	Moderate	–	–	RR 0.90 (0.64–1.27)	Very low
Grade 3/4fatigue	RR 1.17 (0.43–3.18)	Low	–	–	–	–
**Muscle & joint soreness**	RR 0.71 (0.30–1.71)	Low	–	–	–	–
**Rash**	–	–	–	–	RR 0.61 (0.10–3.73)	Very low
Papule and purulent herpes	–	–	–	–	RR 1.60 (0.49–5.25)	Very low
**Fever**	–	–	–	–	RR 0.95 (0.48–1.88)	Very low
**Thromboembolism**	–	–	–	–	RR 0.48 (0.07–3.19)	Very low
**Hyponatremia**	–	–	–	–	RR 1.28 (0.71–2.29)	Very low
Grade 3/4 hyponatremia	–	–	–	–	RR 0.48 (0.10–2.19)	Very low
**Pain**	–	–	–	–	RR 1.00 (0.37–2.67)	Very low

^a^ The evidence quality was evaluated by GRADE. Moderate: we are moderately confident in the effect estimate (the true effect is likely to be close to the estimate of the effect, but there is a possibility that it is substantially different); Low: our confidence in the effect estimate is limited (the true effect may be substantially different from the estimate of the effect); Very low: we have very little confidence in the effect estimate (the true effect is likely to be substantially different from the estimate of effect)

^*^ A statistically significant difference exists (*P* < 0.05)

## Discussion

Proangiogenic factors are usually overexpressed in tumors, resulting in angiogenic switch. Endostatin inhibits tumor angiogenesis through a variety of angiostatic activities on endothelial cells [27]. One potential mechanism is that endostatin inhibits matrix metalloproteinases (MMPs), which mediate proteolytic degradation of the extracellular matrix that can facilitate endothelial cell migration and invasion during angiogenesis [28, 29]. Another mechanism proposes that endostatin binds to α5- and αv-integrins [30, 31], with three major downstream effects: actin disassembly through Src-dependent-p190RhoGAP activation [32], inhibition of the FAK/Ras/p38-MAPK/ERK signaling cascade via α5β1-integrin binding [33], and down-regulation of β-catenin dependent on Wnt signaling [34]. In addition, endostatin binds to vascular endothelial growth factor receptor 2 (VEGFR2) directly without binding to its ligand, inhibiting VEGF-induced phosphorylation and suppressing VEGF-mediated downstream signaling pathway [35, 36].

Patients diagnosed with lung cancer usually experience significant medical service utilization [37]. In clinical practice, IIV of Endostar has increased intravenous admixture workload of nursing staff, as Endostar should be diluted in 500 mL of normal saline daily for a duration of 14 days in each treatment cycle. In addition, NSCLC patients need to lie in bed for 3–4 h each day, hence it is difficult for them to move freely, causing more inconvenience to daily life. The CIV delivery method of Endostar can overcome aforementioned drawbacks by using a portable infusion pump, after preparation of a single admixture for each treatment cycle. Besides, the automatic pump for continuous infusion can usually accurately control the infusion rate and the amount of infusion, alarm abnormalities such as air bubbles, empty fluid, and infusion tube blockage, thus help to reduce the risk of medical errors [25]. Currently, both CIV and IIV of Endostar are administered for hospital inpatients in clinical practice in China. As seen in our systematic review, the duration of administration by CIV was usually shorter (less than 10 days in most studies) than II (usually 14 days in one treatment cycle). Two studies [18, 25] confirmed that CIV of Endostar reduced total volume of infusion, and shortened hospital stay.

Our scoping search before this systematic review indicated that a very small number of potentially relevant RCTs had been conducted previously, and survival outcomes or safety outcomes were poorly addressed. Furthermore, the role of post-approval observational studies in comparative effectiveness research has gained increasing attention in recent years. Therefore, it had been decided to conduct a systematic review and meta-analysis including both RCTs and non-randomized studies, to compare the efficacy and safety of different delivery methods of Endostar. Specifically, NRCTs and cohort studies were included as they are more likely to provide unbiased results than other non-randomized study designs. The inclusion of non-randomised studies have posed several challenges for the design and conduct of this systematic review. First, non-randomized studies are usually poor indexed with inconsistent use of study design labels [38], therefore, one comprehensive search strategy was applied in this systematic review to avoid missing any eligible studies. The second challenge arose in the assessment of risk of bias, as different study designs were included. Downs and Black Checklist can be used to evaluate both randomized and non-randomized studies. Additionally, it was considered to be one of the most valuable tools for evaluating the quality of non-randomized studies [15]. There are 27 items in the checklist distributed between four scales: reporting (ten items), external validity (three items), internal validity (thirteen items) and power (one item). Only the internal validity scale was used for assessing the risk of bias, as generally external validity, reporting or power are unlikely to have direct implications for risk of bias [38]. Third, evidence from NRCTs were not clearly considered or included in the GRADE approach [16]. To be conservative, NRCTs without special strengths or important limitations were judged to provide low quality evidence in this systematic review, as cohort studies were. Fourth, it was possible that different qualities of evidence might have resulted from different study designs addressing the same outcome, as GRADE approach was performed according to different types of study. When this happened (which could be seen for short-term efficacy outcomes in Table 2 and several adverse outcomes in Table 3), estimates from the highest level of quality were adopted and reported as the best available evidence, based on epistemological rationale of evidence-based medicine and principles of GRADE system [16, 39]. Usually higher hierarchy of evidence comes from pooled results of randomized controlled studies, however, there are times when high confidence has been attached to the estimates of effect from non-randomized studies (depending on quality rating results) [40].

This systematic review and meta-analysis found no significant differences between CIV and IIV in short-term efficacy outcomes (ORR and DCR). In terms of survival outcomes, however, very low quality of evidence supported the survival benefit associated with CIV in both OS and PFS compared with IIV. The half-life of Endostar in human body is only about 10 h [41]. Theoretically, IIV can make the concentration of Endostar fluctuate greatly, which means the effective concentration can only act on the tumor tissue in a short time. Instead, CIV can realize the delivery of various solutions or suspensions at a constant rate for days and even weeks maintaining the effective concentration of Endostar [12], which may explain why CIV of Endostar might improve survival outcomes over IIV. Large RCTs with long-term follow-up are needed to definitely demonstrate the efficacy of CIV in comparison with IIV of Endostar.

As for safety outcomes, moderate quality of evidence demonstrated that compared with IIV, CIV of Endostar reduced the risk of myelosuppression (RR 0.55, 95% CI 0.32–0.96) and cardiovascular toxicity (RR 0.21, 95% CI 0.06–0.78) by 45 and 79%, respectively. Especially, the reduction in the risk of cardiovascular toxicity was substantial. Very low quality evidence suggested that CIV might decrease the risk of laryngeal hemorrhage (RR 0.27, 95% CI 0.09–0.84). For all the other adverse events (including all the grade 3/4 adverse reactions), the vast majority of evidence was of low or very low quality, and identified no differences between CIV and IIV. Proteinuria, injection site-related adverse events and phlebitis were not investigated in this systematic review, as they were reported in none of the included studies. It is speculated that by adopting CIV, the amount of drug pumped per unit time is less and the fluctuation of drug concentration is mild, thus it has probably less adverse impact on bone marrow and cardiovascular system. Previous studies also supported that CIV of Endostar could reduce drug toxicity [41].

Lung cancer is one of the major contributors to cancer-caused DALYs in most countries, and has imposed a substantial disease burden to global public health [42, 43]. China has experienced a noteworthy increase in the relative disease burden caused by lung cancer, with 12% of total DALYs from cancers in 1990 to 20% in 2008 [43], and is now facing up with severe predicament of lung cancer burden [37]. Currently, Endostar has been widely used in combination with first-line chemotherapy for advanced NSCLC in China. Its use has been recommended by National Health Commission of China [44] and Chinese Society of Clinical Oncology [45], and has been covered by National Health Insurance of China. With the increasing off-label use of CIV of Endostar in treatment of NSCLC, it is essential to get better knowledge of the relative efficacy and safety of CIV versus IIV to guide clinical practice. Our study has provided current best available evidence for this important clinical question. With scientific and rigorous methods employed, the results indicated that CIV had similar short-term efficacy and lower risk of certain adverse outcomes, and suggested possible survival benefit compared with IIV. Given the fact that survival benefit associated with CIV has not substantiated by high quality of evidence, we argue that advisable caution should be given in clinical off-label use of CIV of Endostar. Future large RCTs with sufficient follow-up, or at least well-designed and executed real-world studies, are warranted to demonstrate the relative survival benefit of CIV.

Our review has three advantages. First, to the best of our knowledge, this is the first systematic review and meta-analysis head-to-head comparing the efficacy and safety of CIV of Endostar with IIV, and the results will inform clinical decision-making in NSCLC treatment. Second, this systematic review included both RCTs and non-randomized studies (NRCTs and cohort studies), and provided an overall evidence profile on the benefit and harm of different delivery methods of Endostar in the treatment of NSCLC. Third, a comprehensive search strategy with high sensitivity was performed, to accommodate the inclusion of non-randomized studies and conduct a search as exhaustive as possible. Our review also suffers from several limitations. First, with a relatively small number of participants in each outcome, imprecision of effect estimates had led to downgrading one or two levels in quality of evidence in all outcomes. Second, due to the small number of studies included (and analysis was not implemented by different histological types in all primary studies), subgroup analysis or meta-regression were not carried out to investigate the influence of histological type, dose, infusion rate and TNM staging on the pooled results. Third, baseline comparability of EGFR status was not assessed or adjusted in most included studies, and this could lead to potential confounding.

## Conclusions

In conclusion, CIV and IIV of Endostar, in combination with first-line chemotherapy, had similar short-term efficacy in patients with pathologically confirmed stage III or IV NSCLC. Compared with IIV, CIV of Endostar reduced the risk of myelosuppression and cardiovascular toxicity substantially. Very low quality evidence supported that CIV could improve both OS and PFS, and large RCTs with long-term follow-up are needed to demonstrate these survival benefits. Advisable caution should be given for off-label use of CIV of Endostar in clinical practice.

## Supplementary information

**Additional file 1.**

## Abbreviations

ADC: Adenocarcinoma
CDSR: Cochrane Database of Systematic Review
CENTRAL: Cochrane Central Register of Controlled Trials
CIV: Continuous intravenous
CPCI: Conference proceedings including Conference Proceedings Citation Index
DALYs: Disability adjusted life years
DCR: Disease control rate
HRs: Hazard ratios
IIV: Intermittent intravenous
MMPs: Matrix metalloproteinases
NRCTs: Non-randomized controlled trials
NSCLC: Non-small cell lung cancer
ORR: Objective response rate
OS: Overall survival
FS: Progression-free survival
RCTs: Randomized controlled trials
RECIST: Response evaluation criteria in solid tumors
RRs: Risk ratios
SFDA: State Food and Drug Administration
SqCC: Squamous cell carcinoma
TTP: Time to progression
QoL: Quality of life
VEGRF2: Vascular endothelial growth factor receptor 2
