# Supplementary material for: Endostar continuous versus intermittent intravenous infusion combined with chemotherapy for advanced NSCLC: a systematic review and meta-analysis including non-randomized studies
Source: BMC Cancer. 2020 Oct 21;20:1021. doi: 10.1186/s12885-020-07527-4 (PMC7579986; doi:10.1186/s12885-020-07527-4)
Supplement: Supplementary file 1 — Additional file 1. [file 12885_2020_7527_MOESM1_ESM.docx]

Additional file 1

**Table S1** Items in the risk of bias assessment tool in this study

| **No.** | **Questions** |
| --- | --- |
| **Q1** | Was an attempt made to blind study subjects to the intervention they have received? |
| **Q2** | Was an attempt made to blind those measuring the main outcomes of the intervention? |
| **Q3** | If any of the results of the study were based on “data dredging”, was this made clear? |
| **Q4** | In trials and cohort studies, do the analyses adjust for different lengths of follow-up of patients, or in case-control studies, is the time period between the intervention and outcome the same for cases and controls? |
| **Q5** | Were the statistical tests used to assess the main outcomes appropriate? |
| **Q6** | Was compliance with the interventions reliable? |
| **Q7** | Were the main outcome measures used accurate (valid and reliable)? |
| **Q8** | Were the patients in different intervention groups (trials and cohort studies) or were the cases and controls (case-control studies) recruited from the same population? |
| **Q9** | Were study subjects in different intervention groups (trials and cohort studies) or were the cases and controls (case-control studies) recruited over the same period of time? |
| **Q10** | Were study subjects randomised to intervention groups? |
| **Q11** | Was the randomised intervention assignment concealed from both patients and health care staff until recruitment was complete and irrevocable? |
| **Q12** | Was there adequate adjustment for confounding in the analyses from which the main findings were drawn? |
| **Q13** | Were losses of patients to follow-up taken into account? |

**Table S2** Risk of bias of included studies

| **Study** | **Q1** | **Q2** | **Q3** | **Q4** | **Q5** | **Q6** | **Q7** | **Q8** | **Q9** | **Q10** | **Q11** | **Q12** | **Q13** | **Total** | **Risk of bias** |
| --- | --- | --- | --- | --- | --- | --- | --- | --- | --- | --- | --- | --- | --- | --- | --- |
| Yao et al. (2018) | 0 | 0 | 1 | 1 | 1 | 1 | 1 | 1 | 1 | 0 | 0 | 1 | 1 | 9 | acceptable |
| Li et al. (2018) | 0 | 0 | 1 | 1 | 1 | 1 | 1 | 1 | 1 | 0 | 0 | 1 | 1 | 9 | acceptable |
| Cheng et al. (2019) | 0 | 0 | 1 | 1 | 1 | 1 | 1 | 1 | 1 | 0 | 0 | 1 | 1 | 9 | acceptable |
| Zhu et al. (2019) | 0 | 0 | 1 | 1 | 1 | 1 | 1 | 1 | 1 | 0 | 0 | 1 | 1 | 9 | acceptable |
| Pang et al. (2015) | 0 | 0 | 1 | 1 | 1 | 1 | 1 | 1 | 1 | 1 | 0 | 1 | 1 | 10 | acceptable |
| Wen and Chen (2013) | 0 | 0 | 1 | 1 | 1 | 1 | 1 | 1 | 1 | 1 | 0 | 1 | 1 | 10 | acceptable |
| Kahaerjiang et al. (2015) | 0 | 0 | 1 | 1 | 1 | 1 | 1 | 1 | 1 | 0 | 0 | 0 | 1 | 8 | acceptable |
| Tang et al. (2015) | 0 | 0 | 1 | 1 | 1 | 1 | 1 | 1 | 1 | 0 | 0 | 1 | 1 | 9 | acceptable |
| Meng (2017) | 0 | 0 | 1 | 1 | 1 | 1 | 1 | 1 | 1 | 0 | 0 | 1 | 1 | 9 | acceptable |
